# Public engagement pathways for emerging GM insect technologies

**DOI:** 10.1186/s12919-018-0109-x

**Published:** 2018-07-19

**Authors:** Michael M. Burgess, John D. Mumford, James V. Lavery

**Affiliations:** 1 W. Maurice Young Centre for Applied Ethics, University of British Columbia, 227 - 6356 Agricultural Road, Vancouver, BC V6T 1Z2 Canada; 2 Centre for Environmental Policy, Imperial College London, Silwood Park Campus, Ascot, SL5 7PY UK; 3 Hubert Department of Global Health, Rollins School of Public Health, and Center for Ethics, Emory University, Atlanta, GA USA

## Abstract

Policy and management related to the release of organisms generated by emerging biotechnologies for pest management should be informed through public engagement. Regulatory decisions can be conceptually distinguished into the development of frameworks, the assessment of the release of a specific modified organism, and implementation decisions such as location and timing. Although these decisions are often intertwined in practice, the negotiation takes place at different stages of technology development and suggests different roles for public engagement. Some approaches to public engagement are more appropriate for different purposes and situations, and it is not always obvious how to go about matching the approach to the purpose. In addition to the diverse technologies involved in generating modified organisms, there are diverse publics with particular interests and different kinds of knowledge. Institutional interests range from commercial development to public regulation and future uptake. Contextual features, such as agency mandates, may limit or structure the extent and approach to public engagement. Different convening groups (government agencies, public interest groups, academics, businesses) and the kind of decision that is being considered determine what kind of input is needed and how the engaging groups will be constituted. This paper considers how the context of the release of genetically modified insects for pest control requires expanding approaches to the design of the public engagement.

## Background

The release of modified insect pests which interact with natural populations of harmful species offers the opportunity to control insect-borne diseases and to control damage to humans, livestock, agriculture and valuable natural environments. Control strategies incorporating genetically modified insects are designed to be specific to particular harmful species, which offers significant environmental advantages to conventional management with more broad spectrum effects. However, the release of modified organisms is also controversial, raising concerns about secondary ecological effects resulting from the sustained elimination or reduction of the harmful insect populations, the possibility of genome-level changes that may have unpredictable effects, and consequences for organic agriculture producers and other stakeholders within and outside the area where modified insects are released. Some concerns arise if genetic controls fail to work as expected while others are relevant if they prove to be more effective than conventional control efforts. While the release of engineered organisms for pest management must meet scientific and regulatory standards, public engagement also has an important role in articulating appropriate objectives and acceptable risks, which can help to guide the design of control programs, reinforce the legitimacy of the strategies that are implemented and build public trust in their governance.

Regulatory decisions about the release of modified pest insects reflect broad, overlapping categories of consideration, including the general acceptability of the concept of genetic control strategies, the authorization of applications for trials of a particular species in a particular place, and ultimately approval, or denial, of implementation at an operational level. Often general guidance is developed to cover these concepts, such as the European Food Safety Authority (EFSA) guidance on environmental risk assessments for GM animals [1] or the broader UK House of Lords [2] report on the use of GM insects. In some cases regulators assess and may approve trials of specific genetically modified insects. Examples of these include decisions in Panama about field trials for GM *Aedes* mosquitoes [3] and New World Screwworm [4], in Florida for field trials of *Aedes* mosquitoes [5], and the trial of the GM Diamondback moth in New York state [6]. In Brazil, full scale commercial release has been implemented for *Aedes* mosquitoes for dengue control in one city [7].

Although it is commonplace to emphasize a role for public engagement across all of these decisions, the objectives, modes of engagement and utilization of public input vary widely. The mandate of a regulatory body may restrict or specify how the public is to be engaged. Engagement related to general guidance may enable a wider range of interests or knowledge to be considered and included, but may make it difficult to articulate risks and benefits in terms that cover the full range of complexity and public concerns without ambiguity or overly technical language. Engagement related to insect control trials or experimental releases makes the range of relevant risks and potential benefits more specific, but many interests or concerns raised by the public may be beyond the scope of a narrow enviromental assessment specified in regulations. Furthermore, citizens may mobilize and seek various forms of engagement with, and accountability from, regulators and implementors, independent of formal regulatory processes and specific regulatory mandates.

This paper considers public engagement in relation to the categories of legislative, regulatory and implementation actions mentioned above. Alternative approaches and objectives are proposed, drawing on the wider literature and experiences with public engagement.

### Public input into frameworks to guide risk assessment

The European Food Safety Authority (EFSA) guidance on GM animals and the UK House of Lords report on genetically modified insects are two examples of high-level guidance about how environmental risk assessment should be carried out and how policy should be formed for modified insects, respectively.

EFSA prepares general guidance documents on aspects of EU Directives and Regulations and each guidance document has a formal public consultation stage. The GM Animals Environmental Risk Assessment Guidance [1] had a two month public consultation period, with written submissions invited to EFSA directly related to the publication of a draft of the guidance document [8, 9]. The GM animal environmental risk assessment guidance document covered mammals and birds, insects, and fish. The insect part of the report received the greatest number of public comments. At the end of the public consultation period, EFSA had received 720 comments across the three categories from 25 interested parties (i.e. institutes, non-governmental organisations, universities, associations, industry organisations, national risk assessment bodies and individuals). There was a very low participation in the consultation from individuals. Strong input came from interested NGOs, some of which was rejected as being outside the terms of reference for the public consultation, which was limited to the specific issues of risk assessment for the environment and public health.

The UK House of Lords enquiry into GM insects [2] invited evidence in person and gave the option for written public submissions. The purpose was to explore the potential of GM insect technologies to control human and livestock diseases and crop pests, and to establish whether the regulatory environment in the United Kingdom was conducive to the development and deployment of GM insect technologies. In addition to the invited witnesses there were 26 written submissions, from individuals and organisations (including some duplication of verbal evidence from the invited witnesses). Most was from public institutions, one from a private company, several from NGOs and several from individuals, and as such, reflected the views of experts and professionals, rather than the broader public.

Electronically submitted written comments directed to EFSA and written reports submitted to the House of Lords were considered in the final drafting of the resulting guidance and report. In the case of the EFSA guidance the consultation was focused on the specific issues addressed in the draft guidance, and the engagement process effectively restricted commentary to the draft report itself. The “public” most likely to respond to such a call are those who have identified their interests in the area, have the ability to understand the document, and are able to dedicate sufficient time for review and response. In this sense the approach to public engagement is really an invitation for established stakeholders, including those with commercial interests and scientific experts, to consider the draft and respond to it. The objective of the engagement is, effectively, to determine the nature and scope of these established stakeholder concerns and which of them to respond to in a revision of the final report. Responses that questioned the EFSA technical mandate, the competence and operation of the guidance process or the scope of concerns imposed by the decade-old Directive were acknowledged, but not addressed in further editing of the guidance [1].

The outcome of the invitation for public submissions to the House of Lords enquiry was similar to the EFSA process in that the approach to public engagement led to a limited and specialized set of responses: six responses from individuals associated with university departments, one response from an insect conservation NGO, and institutional responses that were broadly positive while recommending case by case review and appropriate regulatory oversight.

It is instructive to note what this kind of formal engagement is not intended to achieve. While draft guidance is made public for consideration, little effort is made to make the draft accessible to a wide readership, since the guidance is ultimately intended for a specialized and technical user group. No effort is made to highlight key points in a more popular summary, for instance. By design, this approach is not intended to inform a wider public, nor is it likely to reassure a wider public or to encourage broad public participation in decisions. EFSA has a mandate to provide technical evidence and advice, not input on potential social or economic implications where wider public involvement might be more relevant. This is different from the “upstream” engagement mandate characterized by the UK House of Lords [10] and promoted by many Science and Technology Studies scholars as capable of identifying values and benefits that are different from the goals of the technology being assessed [11]. The EFSA approach is unlikely to incorporate respect for divergences of perspectives or open up socially deliberated evaluative criteria [12]. The design of the EFSA consultation process is informed, to a large extent, by considerations of feasibility and efficiency. The communication is written intentionally for a narrow audience and the soliticed responses are restricted to written reactions to the draft document. As such, it is important to recognize that the process would not provide EFSA with an enhanced understanding of how a wider public would make the inevitable compromises and trade-offs in decisions that will be shaped by the technical opinions resulting from application of the guidance.

The EFSA and similar approaches to public engagement serve to put established stakeholders and experts on notice of the scientific assessments that the authorities have made and collect any additional input that might affect technical components of environmental risk assessments. They do not invite deeper and broader engagement with the underlying values and trade-offs that are embedded in the draft documents, ruling any such engagements out of scope. They presume that citizens who are not experts or technical stakeholders do not need to be consulted because the kind of knowledge that the authorities want is related specifically to technical information and assessments at this stage. Efforts to understand the wider public interest may be deferred to later specific implementation decisions where public values can be applied to particular cases and the plausibility of risk pathways can be addressed more explicitly.

### Assessments and implementation decisions

Decisions to approve individual trials of modified insects or to release them in control programs move beyond high-level guidance to the assessment of whether the balance of risks and benefits is in the public interest. Although the risks and benefits specific to a given release are more explicitly available for comparisons in trade-off decisions, the stakeholders involved in these decisions often have considerable investment in specific outcomes. There may also have been choices that preclude previously available pest control alternatives, for example removing some insecticides from registration approval, making it more difficult to identify and challenge these commitments once the decision process has moved to a specific application.

A recent case that illustrates these points is the Florida Keys *Aedes* mosquito case, involving trial release of male mosquitoes with an introduced genetic trait that prevents offspring maturing beyond the larval stages. This example demonstrates how specific decisions about whether to release modified species are often tied to implementation decisions such as where the release should be initiated. But the conceptual distinction is important for consideration of how and what public should be consulted.

*Aedes aegypti* mosquitoes, an introduced species in the Western Hemisphere, are responsible for the spread of dengue, Zika, chikungunya and yellow fever viruses. Outbreaks can occur at relatively low densities of mosquitoes, particularly because mosquitoes breed in close proximity to human dwellings. The release of the GM *Aedes* in Panama, Brazil and the Cayman Islands has demonstrated a decline in the target mosquito populations, and the company responsible now claims some resulting declines in disease transmission [13].

For the Florida trial, a county-wide public referendum was held in conjunction with national elections in November 2016. A majority across the county (58%) supported the GM *Aedes* trial release, but in the proposed initial release site, Key Haven, a majority (65%) voted against the release, prompting the public mosquito control authorities to move the proposed release to an alternative site within the county. The public referendum asked a simple question: (Yes/No) *Are you in favor of the Florida Keys Mosquito Control District conducting an effectiveness trial in Monroe County, Florida, using genetically modified mosquitoes to suppress an invasive mosquito that carries mosquito-borne diseases?* Alternatives or implications were not clearly identified in the referendum, for example whether to move the site, delay the trial, seek specific additional information, or undertake any specific alternatives.

The chairman of the Florida Keys Mosquito Control District Board of Commissioners said he opposed the referendum because the Board was elected to make these kinds of hard choices “from a position of knowledge, and not emotion.” “The opponents have very little information, and they are led by a few people who are non-science-based… We have tried to explain the real answers to them. They are not interested in the truth.” [14]. The same report interviewed a local resident who said that Zika did not scare her as much as scientists tinkering with animals. Like others, she fears that some of the genetically modified female mosquitoes (scientists expect a small percentage to be released inadvertently) will bite people and cause unexpected consequences. Some opponents disputed the risk of disease, since no cases of Zika had occurred in the area and no dengue cases had been reported in recent years. Others doubted the need for specific *Aedes* control measures because other control efforts, with pesticides and breeding site elimination, would continue to be intensively applied against the several native mosquitoes that cause significant nuisance biting in south Florida. So debate ranged from technical issues around the ecological and health impacts to questions of how the public mosquito control need was framed and justified and how personal values should be represented by elected officials.

This example of public engagement by non-binding referendum seeks politically legitimate input from the public. The opportunity for the general public to vote on the trial has the advantages of visibility and accessibility over the public posting for commentary approach taken by agencies such as the EPA or USDA in granting approvals for specific trials or general use. This type of public input is particularly relevant as decisions approach operational implementation by public authorities at public risk and expense. The vote provides decision-makers with information about the tolerability of the proposal, and about how this tolerability is distributed geographically among the voting districts of the county, but it does not afford the public an opportunity to express a choice amongst alternatives. Referenda cannot offer complex choices or provide balanced information on the ballot paper, and often leave a divided public with different interpretations of the options perceived to be relevant.

The decision to move the trial to another site demonstrates that the opposition of voters in Key Haven was given weight by decision makers and that local public acceptance of a trial was an important criterion for site selection.

The New York Times report of the referendum illustrates a common tension when the wider public is given the opportunity to participate directly in public decisions [14]. The chairman of the Florida Keys Mosquito Control District Board of Commissioners expressed concern that public input is poorly informed, emotional, and led by a few persuasive people who are not scientific experts. The resident quoted by the New York Times expressed concerns that demonstrate a lack of trust in the experts, and skepticism about their portrayal of the relative risks.

### Public engagement for trustworthiness and legitimacy

Public engagement can be understood as any activity that seeks to inform the public, collect information from the public or enable public participation in decisions, policies or practices. How “public” is understood might be as variable and as important as the range of approaches to engagement. A public engagement process may provide some information to the public as well as gather views from the public, both in response to information provided or from public understanding that is independent of any information related to the engagement. The information content and the way it is presented to the public often shapes the outputs of the response from that public. The outputs of an engagement might be new information to be considered by the organizers or clients, or it could be a collaboration on recommendations or advice. Outputs could also be aggregates of individual opinions, themes identified by analysts, or collective conclusions of the participants.

Many public engagements are characterized as “participation” or “deliberation.” These kinds of public engagement tend to emphasize a role for the public to participate or collaborate in making decisions or recommendations. As such they involve an exchange of information to bring the participants into a level of knowledge that enables them to provide informed and useful advice or participate in decisions. Arnstein’s ladder of citizen participation [15] and more recent work on a typology of public engagement by Rowe and Frewer [16] distinguish participatory public engagement characterized by a two-way flow of information and a kind of partnership or sharing of power from other consultative or communication oriented engagement. Other informative classification frameworks have also been proposed: the International Association of Public Participation Federation “Public Participation Spectrum” [17], one based on ideal types derived from interviews of science researchers [18], and another on the different “imperatives” that engagements presume or on which they are assessed [12].

Abelson and colleagues have suggested four characteristics that can be used to evaluate deliberation [19]. These same dimensions can be used to consider the different ways in which a public engagement can be organized. The first is how the public is represented in selection and recruitment. The second is how the process or procedures are structured. The third is what information is used in the engagement. The fourth is the outcomes and decisions arising from the engagement. Each of these components involve considerations that are essential for public engagements to achieve the promise of appropriate public involvement and influence.

Representation in public engagement is not a simple statistical notion, but a complex set of considerations about how the participants in an engagement will be selected, recruited and reflect the range of interests and perspectives that are important to the issues being considered. All members of the general public have legitimate interests in the issues and decisions, even if they have not yet considered them and determined their position on the decisions. Some members of the public have considered their interests and may have associated in groups that are invested in the promotion of a particular interpretation of the public interest. Sometimes unfortunately referred to as “disinterested” or “naïve” publics, the general public who have not yet formed settled opinions or formed allegiances might be in a better place to openly consider the cases made by those with investments, whether as stakeholder members of the public, or those with financial, professional or political commitments.

The engagement process typically combines the provision of information, directing attention to key issues, and generating input or recommendations. Information can include technical, regulatory and social aspects. Experts, regulators and stakeholders often play an important role, whether by being drawn on to provide summary materials, or directly as observers or participants in the public engagement [20]. Public engagement must carefully consider how to organize the process of engagement so that it is perceived as legitimate to those whose interests are represented and who receive the input or recommendations, while avoiding undue influence by experts or stakeholders.

The way in which discussion is directed, whether by a facilitator, a discussion guide or a process like scenario planning [21] is also an important part of the process. Public engagement processes inevitably frame the discussion, and must explicitly consider and design how the framing shaped the process and outputs [22, 23].

A successful public engagement will have demonstrable outcomes and decisions, which in turn should derive enhanced legitimacy from the public engagement. Participants are more likely to experience the engagement as legitimate if there are perceptible outcomes. The outcomes of a deliberation can include the conclusions that are drawn during the event as well as through subsequent analysis [24]. We should not expect consensus, certainly on all issues, and persistent disagreements may be as informative for decisions as convergence of opinions. The expectation of a deliberative engagement would be that the conclusions reflect a “civic-minded” group decision in the sense that it reflects what the group thinks is appropriate given the differences of individual perspectives of the participants and the wider social context [25]. Deliberative democracy theorists suggest that the deliberation enables participants to generate conclusions that the wider public would generate if they were informed and civic-minded [26]. Implementation of the outcomes into decisions depends on the context, political will and what other societal interests need to be considered by those who have the related mandates.

### Trustworthiness and legitimacy in decisions related to modified pests

Negotiating whether, how and under what circumstances to release modified insects inevitably requires making judgments about what is in the best interests of citizens as a whole. There is no reason to presume that there is a single, unitary answer to what is in the public interest, and recognizing persistent disagreement and how to incorporate that in governance is very important [24, 27]. The decisions based on these assessments are made in the context of the influence of the relevant industrial and political interests, and what risks or benefits count the most will be contentious, with considerable diversity of public perspectives. Decisions that merely present expert opinion for comment are unlikely to stimulate wider public engagement, and the framing of the decisions as technical matters also discourages wider consideration and participation. Citizens are aware of the influence of industry and NGOs and the technical capacity of regulators, and may feel their own limitations in both influence and technical knowledge. In this context, decisions might fail to be trustworthy due to skepticism about whether the wider range of public interests have been acknowledged and reflected in the decisions. This distrust is not likely to be remedied by more scientific explanation, although a focus on establishing criteria for trustworthy governance, including confronting concerns about commercialization, might begin to define conditions of trustworthy governance [11, 20, 28].

Public engagement that provides relevant technical information, fair acknowledgement and consideration of the interests of all stakeholders, and provides a genuine opportunity for all, or at least a diversity of citizens, to have input is more likely to introduce a wide range of public interests and consideration. When combined with scientific risk assessment and stakeholder engagement, such activities provide decision makers with a better basis from which to decide what is really in the interests of a diverse public. Further, deliberative public engagement can provide decision makers with advice or recommendations from an informed, diverse group of citizens that is oriented to negotiating how to make decisions given controversy and diversity. This kind of public engagement is more likely to produce trusted decisions that are seen as legitimate, and may open opportunities for compensatory actions that balance gains and losses to particular groups.

When the objective is to develop a framework or “points to consider” for assessing the appropriateness of deploying genetically modified insects in pest control, the general nature of the question anticipates considerations of different values and goals, which may contribute to more inclusive or participatory governance [29–31]. The elicitation of broader values and objectives may seem out of scope to narrow mandates, which often focus on technical approaches to risk management, and may be difficult questions to articulate in specific terms, compared to the details that often become clearer in later implementation decisions. Experts and stakeholders are more likely to develop clear positions about specific issues and concerns than members of the general public, who may not yet have formulated set views nor considered their own related interests. Under these circumstances input from the broader public is likely to ensure that the range of issues considered is sufficiently broad and comprehensive. Whereas for implementation decisions “local knowledge” is more likely to provide insights about considerations that are otherwise not obvious. Members of the public with experience in the activities and regions where releases are likely to occur are most likely to have useful insights at this level. But general invitations to comment may be inadequate, and specific invitations to participate on panels that provide information and are open to input from the public members might be a preferred approach.

As discussed above with the example of the Florida referendum on *Aedes aegypti* mosquitoes, assessments of whether to release specific modified species at a particular location are often intertwined with practical implementation decisions. This conflates the issues of whether release is justified with how to negotiate who will most directly bear the effects of a specific release.

A broader public engagement in the assessment of whether to release modified pests would make decisions more legitimate by enhancing the inclusiveness and openness of the decision making process, irrespective of the decisions themselves. When there is a particular technology to assess, the trade-off between intended benefits and the range, nature and uncertainty of risks can be considered. This makes it possible for a wider public, given appropriate support, to participate in these assessments. Further, at this point of assessment, vested interests may have developed, with industry and possibly research interests aligning with release and opposing public interest groups aligning in favour and against release. It is therefore important to involve members of the public who are uncommitted in the assessment, and who can consider collective and diverse interests and perspectives. It is here that broad public engagement, perhaps using deliberative engagement approaches, might be most useful. The Australian Science and Technology Engagement Pathways has “rules of engagement” and “intervention pathways” that might be useful in the design of such approaches [32, 33]. But as several analysts recognize, there is no universal approach and each public engagement must struggle to find a way to authentically reflect diverse public views, but to do so in a way that resonates with the relevant decision makers [32, 34].

The mosquito referendum emphasized the implementation aspect of the trial release and enabled citizens who would be affected by the release to express their interests, perhaps in a classic “NIMBY” *(not in my backyard)* reaction. Nevertheless, when an implementation decision is being considered, the issues move from the assessment of whether a release is justifiable to who will receive the effects, positive and negative. It is important to directly consult with members of the public who believe they will be directly affected. Here the public is not expected to be non-partisan, but to act in an informed manner to consider their own interests and those of others.

The different engagements of the public, with diverse objectives, combine to make the decisions more worthy of being trusted by citizens, and by those with strong, direct interests. Finally, an additional objective is to make translation of decisions to actual practice more sustainable, through having the public, consumers, producers and regulators jointly involved in the process. The final section considers the features of public engagement that are important for progressing toward, and achieving, these objectives.

The transparency and responsiveness of the final decisions to the public advice is also important. While advice from these public deliberations may not be binding in a straightforward manner, once articulated there needs to be accountability for how they are engaged in the decision making process. Participants are likely to have more confidence in the process if they believe their interests have been acknowledged and considered. Together, the public deliberation and transparent accountability for how the advice was considered will increase the legitimacy and trustworthiness of the decisions.

## Conclusions

A structural challenge is that few, if any, government agencies have a sufficiently wide mandate for public engagement to support the resource intense activities discussed earlier. Occasionally, special commissions may be formed by governments to include a wide mandate for public engagement. The Danish Board of Technology had a longstanding mandate that lasted several decades [35]. More commonly, intense public engagement exercises are funded as research or special projects. A wide range of projects are described on a website, Participedia [36].

Decisions related to the release of modified insects will be made in a context where what benefits are to be considered, and how trade-offs are to be made and releases implemented, are contested. In this context, it is very difficult to produce decisions that are trustworthy and legitimate, and that reflect consideration of a wide range of public interests. Even with a mandate to engage the publics, government agencies rarely have the resources to appropriately engage a wider public. Further, while public enagement is a crucial component of making decisions that considers the range of relevant public interests, industry and stakeholder groups, academic social science researchers, and NGOs also have important contributions in understanding diverse public interests.

Resourcing the appropriate public engagement for each kind of regulatory decision, and then structuring it so that it is effective and trusted, should be included in high level decision frameworks and for decisions about field trials and release of genetically modified insects. Guidance on overall decision frameworks, and maybe even the legislation itself, should commit to public engagement. Seeking public input only on whether and where to release insects precludes consideration of the goals of release and how the long term effects will be monitored and regulated.

The Australian Science and Technology Engagement Pathways framework provides detailed guidance on a scaleable approach to public engagement, while some of the participants in their formation caution that actual engagements need to be contextually relevant [32, 33]. Despite a wide critical literature, Chilvers suggests that there is still much to be gained through public engagement:

At a substantive level constructivist work in the sociology of scientific knowledge (SSK) and public understanding of science (PUS) has developed powerful epistemological and ontological arguments claiming that exposure to a wider range of public knowledge, values, and meanings can create science that is more socially intelligent and robust [29].

It is probably a mistake to consider public engagement as a way for modified insects in pest control to avoid the public conflicts experienced around GM food and agriculture. The lesson from that era is the presumption that the question was primarily about risks that could be identified by regulatory science [11]. The way forward for public engagement related to modified pests is to recognize that public engagement is one of many important inputs in a more open approach to define the acceptable goals of the release of modified insects for pest control, and to include members of the public in the governance in a way that demonstrates trustworthiness in a context of economic, political and social complexity. As Stirling put it:

Only in this more open-ended fashion may we realistically hope to achieve a richer, wider, and more vibrant empowering of human agency in the deliberate social choice of technological futures [12].

## References

[1] European Food Safety Authority. Guidance on the environmental risk assessment of genetically modified animals EFSA Journal 2013; doi:10.2903/j.efsa.%202013.3200. 10.2903/j.efsa.2010.1879 PMC13129916 42079552

[2] House of Lords Science and Technology Select Committee. Genetically modified insects. HL Paper 68. 2015. https://www.publications.parliament.uk/pa/ld201516/ldselect/ldsctech/68/68.pdf. Accessed 6 Aug 2017.

[3] Gorman K, Young J, Pineda L, Márquez R, Sosa N, Bernal D, Torres R, Soto N, Lacroix R, Naish N, Kaiser P, Tepedino K, Philips G, Kosmann C, Cáceres L (2016). Short-term suppression of Aedes aegypti using genetic control does not facilitate Aedes albopictus. Pest Manag Sci.

[4] Concha C, Palavesam A, Guerrero FD, Sagel A, Li F, Osborne JA, Hernandez Y, Pardo T, Quintero G, Vasquez M, Keller GP, Phillips PL, Welch JB, McMillan WO, Skoda SR, Scott MJ. A transgenic male-only strain of the new world screwworm for an improved control program using the sterile insect technique. BMC Biol 2016; doi:10.1186/s12915-016-0296-8. 10.1186/s12915-016-0296-8 PMC5004303 27576512

[5] Atkins K. In Florida keys, most voters favor GMO mosquito release experiment. Miami Herald Nov 8. 2016. http://www.miamiherald.com/news/politics-government/election/article113478728.html Accessed 6 Aug 2017.

[6] Animal and Plant Health Inspection Service, United States Department of Agriculture. Proposal to permit the field release of genetically engineered diamondback moth in New York. Enviroinmental Assessment. 2014; https://www.aphis.usda.gov/brs/aphisdocs/13_297102r_dea.pdf Accessed 6 Aug 2017.

[7] “Aedes do bem” reduz em 80% risco de doenças em Piracicaba. Exame 30Mar. 2017; http://exame.abril.com.br/brasil/aedes-do-bem-reduz-em-80-risco-de-doencas-em-piracicaba/ Accessed 6 Aug 2017.

[8] European Food Safety Authority. Outcome of the public consultation on the draft scientific opinion of the scientific panel on genetically modified organisms (GMO) providing guidance on the environmental risk assessment of genetically modified animals. Supporting Publications 2013; doi:10.2903/sp.efsa.2013.EN-428. 10.2903/j.efsa.2010.1880 PMC13129908 42079532

[9] Mumford JD, Devos Y, Liu Y, Mestagh S, Waigmann E (2016). EFSA guidelines on environmental risk assessment of GM animals, including insects. IOBC/WPRS Bulletin.

[10] House of Lords, Select Committee on Science and Technology (2000). Science and society – third report.

[11] Wynne B (2006). Public engagement as a means of restoring public trust in science--hitting the notes, but missing the music?. Community Genetics.

[12] Stirling A (2008). “Opening up” and “closing down”: power, participation, and pluralism in the social appraisal of technology. Sci Technol Hum Values.

[13] Joseph A. Florida keys voters split on genetically modified mosquito trial. STAT 8 Nov 2016; https://www.statnews.com/2016/11/08/florida-keys-voters-split-on-genetically-modified-mosquitoes/ Accessed 6 Aug 2017.

[14] Alvarez L. In Florida keys, some worry about ‘science and government’ more than Zika. New York Times 24 Aug 2016; https://www.nytimes.com/2016/08/25/us/zika-florida-keys-mosquitoes.html?_r=1 Accessed 6 Aug 2017.

[15] Arnstein SR (1969). A ladder of citizen participation. J Am Inst Plann.

[16] Rowe G, Frewer LJ (2005). A typology of public engagement mechanisms. Sci Technol Hum Values.

[17] International Association for Public Participation. Public Participation Spectrum. 2015; http://iap2canada.ca/page-1020549 Accessed 9 Aug 2017.

[18] Marks NJ (2013). Six ideal types of public engagement with science and technology: reflections on capital, legitimacy and models of democracy. Int J deliberative Mech Sci.

[19] Abelson J, Forest PG, Eyles J, Smith P, Martin E, Gauvin FP (2003). Deliberations about deliberative methods: issues in the design and evaluation of public participation processes. Soc Sci Med.

[20] Burgess MM (2014). From ‘trust us’ to participatory governance: deliberative publics and science policy. Public Underst Sci.

[21] Schoemaker PJH (1993). Multiple scenario development: its conceptual and behavioral foundation. Strateg Manag J.

[22] Druckman JN, Nelson KR (2003). Framing and deliberation: how citizens’ conversation limit elite influence. Am J Polit Sci.

[23] Walmsley HL. Mad scientists bend the frame of biobank governance in British Columbia. J Public Deliberation. 2009;5(1) http://www.publicdeliberation.net/jpd/vol5/iss1/art6/ Accessed 6 Aug 2017.

[24] O'Doherty KC. Synthesising the outputs of deliberation: extracting meaningful results from a public forum. J Public Deliberation. 2013;9(1). Article 8. Available at: https://www.publicdeliberation.net/jpd/vol9/iss1/art8. Accessed 6 Aug 2017.

[25] O’Doherty KCAKH, Burgess MM (2012). Involving citizens in the ethics of biobank research: informing institutional policy through structured public deliberation. Soc Sci Med.

[26] Goodin RE, Dryzek JS (2006). Deliberative impacts: the macro-political uptake of mini-publics. Politics Soc.

[27] Chilvers J (2013). Reflexive engagement? Actors, learning, and reflexivity in public dialogue on science and technology. Sci Commun.

[28] Aitken M, Cunningham-burley S, Pagliari C (2016). Moving from trust to trustworthiness: experiences of public engagement in the Scottish health informatics Programme. Sci Public Policy.

[29] Chilvers J (2008). Deliberating competence: theoretical and practitioner perspectives on effective participatory appraisal practice. Sci Technol Hum Values.

[30] Delgado A, Kjølberg KL, Wickson F (2011). Public engagement coming of age: from theory to practice in STS encounters with nanotechnology. Public Underst Sci.

[31] O’Doherty KC, Burgess MM, Edwards K, Gallagher R, Hawkins A, Kaye J, McCaffrey V, Winickoff D (2011). From consent to institutions: designing adaptive governance for genomic biobanks. Soc Sci Med.

[32] Marks NJ, Russell AW (2015). Public engagement in biosciences and biotechnologies: reflections on the role of sociology and STS. J Sociol.

[33] Russell AW (2013). Improving legitimacy in nanotechnology policy development through stakeholder and community engagement: forging new pathways. Rev Policy Res.

[34] Hennen L (2012). Why do we still need participatory technology assessment?. Poiesis Prax.

[35] Horst M (2014). On the weakness of strong ties. Public Underst Sci.

[36] Participedia. https://www.participedia.net Accessed 6 Aug 2017.

